# Nutritional Assessment of Polish Middle-Distance Runners: Analysis of Biochemical Parameters and Antioxidant Status—Pilot Study

**DOI:** 10.3390/biology15100737

**Published:** 2026-05-07

**Authors:** Agnieszka Chrustek, Anna Przybylska, Oliwia Pakuła, Anna Proszowska, Anna Filarecka, Agnieszka Dombrowska-Pali, Dorota Olszewska-Słonina, Marcin Koba

**Affiliations:** 1 Department of Pathobiochemistry and Clinical Chemistry, Faculty of Pharmacy, L. Rydygier Collegium Medicum in Bydgoszcz, Nicolaus Copernicus University in Torun, M. Curie-Skłodowskiej 9 Street, 85094 Bydgoszcz, Poland; annafilarecka@op.pl (A.F.); dorolsze@cm.umk.pl (D.O.-S.); 2 Department of Toxicology and Bromatology, Faculty of Pharmacy, L. Rydygier Collegium Medicum in Bydgoszcz, Nicolaus Copernicus University in Torun, A. Jurasza 2 Street, 85089 Bydgoszcz, Poland; annaprzybylska00@gmail.com (A.P.); ikiaro16@gmail.com (O.P.); annaproszowska@cm.umk.pl (A.P.); kobamar@cm.umk.pl (M.K.); 3 Department of Perinatology, Gynecology and Gynecologic Oncology, Faculty of Health Sciences, Collegium Medicum in Bydgoszcz, Nicolaus Copernicus University, Łukasiewicza 1, 85-821 Bydgoszcz, Poland; agnieszka.pali@cm.umk.pl

**Keywords:** oxidative stress, middle-distance runners, biochemical parameters, vitamins, micro- and macronutrients

## Abstract

This study aimed to examine the nutrient intake in Polish middle-distance runners. The secondary objective was to examine the biochemical profile and antioxidant status in the blood serum of the runners, and then compare to the control group. The research was conducted among 44 volunteers. Information regarding participants’ diet was gathered for three days before the study took place. Also, blood serum of athletes and control subjects was examined to obtain information about their antioxidant status, biochemical parameters, and micro- and macronutrients. This study showed that runners consumed more protein, sodium, zinc, phosphorus, water, vitamin A and E than the control group. The daily intake of selected vitamins, micro- and macronutrients in a group of athletes is approximately two times higher than in the control group. The control group consumed more polyphenols compared to the runners group (*p* < 0.05). The consumption of phenols was almost two times lower among runners compared to non-runners. The higher uric acid concentration (*p* = 0.025) and lower chloride concentration (*p* = 0.011) were observed in the blood serum of runners compared to the control group. The diet of middle-distance runners is well-balanced but need some modifications, especially regarding the intake of vitamin E, folates and polyphenols.

## Simple Summary

Nutrition plays a significant role in post-exercise recovery. This study aimed to assess the nutritional status of Polish middle-distance runners and to analyze the biochemical parameters and antioxidant status of the runners’ serum. The nutritional habits of 44 middle-distance runners from Poland were assessed using the Diet 6.0 program. The impact of diet and exercise on serum biochemical parameters was also analyzed. Polish middle-distance runners have been observed to consume adequate amounts of energy, protein, carbohydrates, micronutrients, macronutrients, and vitamins. The exception is a lower intake of vitamin E, folates, and polyphenols.

## 1. Introduction

Proper nutrition plays a crucial role in the proper functioning of the human body, supporting physical activity, improving athletic performance, and supporting post-exercise recovery. Professional runners are expected to meet their nutritional needs by consuming food of appropriate quality and quantity [1]. Incorrect nutrition can negatively affect runners’ performance by reducing lean body mass, impairing immune function, increasing susceptibility to injuries and causing symptoms associated with overtraining [2].

Maintaining peak performance largely depends on optimal energy intake. Low energy intake can negatively impact runners’ endocrine, circulatory, immune, metabolic, reproductive, and digestive systems [3]. High carbohydrate intake can improve performance during training by increasing the availability of exogenous carbohydrates and glycogen storage in muscle and liver [4]. The intake of protein and amino acids by an athlete is crucial for the rebuilding and regeneration of skeletal muscle [5]. It is recommended to consume fat between 20% and 35% of the total calorie intake, with saturated fat intake being less than 10% [6]. The consumption of vitamins, microelements (manganese, copper, zinc, iron) and macroelements (phosphorus, calcium, potassium, sodium) is also crucial for an athlete’s performance. The physiological daily requirement for microelements is 20 mg, while for macroelements it is 100 mg [7]. Proper electrolyte, mineral, and vitamin balance plays a vital role in maintaining antioxidant status.

Reactive oxygen species, nitrogen species, and recently identified reactive sulfur species (RSS) cause oxidative damage to lipids, proteins, and DNA. More importantly, they may be free radicals or non-radical oxidants [8]. Free radicals play an important role in the proper course of many life processes, e.g., they regulate gene expression and concentration of calcium in cells, and eliminate microorganisms from the body [9,10,11]. Moreover, reactive oxygen species (ROS), such as superoxide anion (O2•−), hydroxyl radicals (•OH), peroxide radicals (ROO•), alkoxyl radicals (RO•) and hydrogen peroxide (H_2_O_2_) can attack biological macromolecules in vivo, which leads to damage to proteins, lipids, and DNA, but also to cell aging and diseases caused by oxidative stress [12].

Antioxidants protect against oxidative stress through the conversion of free radicals into non-radicals, thereby contributing to the reduction in their reactivity or preventing their transformation from inactive radicals into more harmful structures [13]. The antioxidant system in the body is an extremely important structure that is responsible for maintaining redox balance. Endogenous and exogenous antioxidants are extremely helpful in this regard [14]. Endogenous antioxidants are involved in physiological processes that have enzymatic activity, such as superoxide dismutase (SOD), catalase (CAT), and glutathione peroxidase (GPx) and non-enzymatic antioxidants, which are molecules capable of quickly neutralizing reactive oxygen species (ROS), such as glutathione, lipoic acid, bilirubin, and ferritin [8]. In turn, attention should be paid to exogenous antioxidants from natural dietary sources. These include fruits, vegetables, fruit and vegetable juices, tea, coffee, nuts, cereal products, and legumes [14,15]. Antioxidant compounds that come from the diet (vitamin E, vitamin C, carotenoids, Zn, Mn, Cu, Se, and polyphenols such as flavonoids, phenolic acids, stilbenes, lignans) influence the activity of endogenous antioxidants, thanks to which they exert a synergistic effect. Many studies show that preventing the occurrence of pathological processes when using diets rich in antioxidants is possible thanks to food rations rich in exogenous antioxidants [14].

Physical activity has a beneficial effect on human health; however, there is evidence that intense exercise increases the level of ROS by disturbing the oxidative–antioxidant homeostasis towards oxidative reactions [16,17]. During exercise, uncontrolled “leakage” of electrons can occur during the transport of electrons along the respiratory chain, as well as the oxidation of hemoglobin [18,19]. Depending on the duration and intensity of training, the risk of oxidative damage increases [17,20,21]. Excess ROS can lead to damage to the structural elements of cells and, as a result, to disorders of homeostasis, as well as death as a result of necrosis or apoptosis. It is believed that free radicals are important during physical activity, but also in the occurrence of cardiovascular diseases, diabetes, and neurodegenerative diseases [9,22]. In order to counteract the negative effects of free radicals, the cells of living organisms have developed an antioxidant defense system based on the action of groups of molecules, so-called free radical scavengers. The antioxidant defense system includes exogenous (low-molecular) antioxidants (i.e., vitamins A, C, E, polyphenols) and endogenous (high-molecular, enzymatic) antioxidants [14,23]. Athletes use a variety of measures to mitigate the adverse effects of exercise and maximize its benefits. Many athletes take antioxidant supplements and follow an antioxidant-rich diet (functional food) to mitigate oxidative stress [24]. Low nutrient intake affects more than just the athlete’s energy status, but also the possibility of a poorer supply of vitamins (B vitamins) used during physical exercise [25]. The nutrition of athletes affects the metabolic processes of the body, but also has a regenerating effect on intense physical effort.

There is a lack of data in the literature regarding the assessment of the intake of antioxidant vitamins, micro- and macronutrients for European middle-distance runners. This study aimed to examine the nutrient intake, including micro- and macronutrients, water-soluble vitamins, and fat-soluble vitamins, in Polish middle-distance runners. The secondary objective was to examine the biochemical profile, micro- and macronutrient content, and antioxidant status in the blood serum of the runners, and then compare this to a group of untrained individuals.

Athletes pay attention to their nutrition, so their diet is balanced, but it may contain deficiencies. The diet of middle-distance runners may contain lower levels of polyphenols and vitamins than recommended. The runners’ serum biochemical parameters will differ from those of the control group. Results will be influenced by the middle-distance runners’ diet and their training. When examining serum biochemical parameters of runners, it is worth examining the influence of additional factors (gender, supplementation) on performance.

## 2. Materials and Methods

### 2.1. Participants

The study involved 44 people aged 21 to 30. Women accounted for a greater percentage (59.09%) than men (40.91%).

The groups included participants who did not smoke, did not take medications or probiotics, and came from cities in the Kuyavian-Pomeranian Voivodeship.

The study group consisted of 22 middle-distance runners (800–1500 m), including 11 women and 11 men, aged 21 to 30 years. The runners were in a training cycle. The training cycle consisted of interval training, an 800 m run, interspersed with rest. All volunteers were asked for a description of their normal lifestyle, in particular, their eating habits. People participating in the study did not follow elimination diets. The inclusion criteria were no hypertension, diabetes, cardiovascular diseases, and other comorbidities. Participants took supplements in the form of creatine monohydrate (1–2 g), vitamin D3 (2000 IU).

The control group included 22 people, including 15 women and 7 men, aged 21 to 30 years. The control group did not take supplements. The inclusion criteria were no hypertension, diabetes, cardiovascular diseases, and other comorbidities. People participating in the study did not follow elimination diets.

All runners participating in the study read information about it, completed the questionnaire, and expressed their conscious opinion, written consent to participate in it.

### 2.2. Samples

Blood was collected in the morning (one-time collection). At the time of blood collection, the runners had just finished their morning training (9–10 a.m.) and were not fasting. Blood samples were collected from venous blood into sterile biochemical tubes with a coagulation activator (silica). After collection, the biological material was centrifuged for 10 min at 3500 rpm and 20 °C. The serum was then pipetted with an automatic pipette and transferred to individual Eppendorf tubes. The material prepared in this way was stored in string-closure bags and at a temperature of −80 °C for no longer than 6 months. The serum preparation method was developed based on the internal procedure of the diagnostic laboratory.

### 2.3. Nutrition Study

Information regarding the participants’ diet was gathered for three days before the study took place. All 132 daily food rations (3 days × 44) written down in the form of menus were collected. The respondents determined the size of consumed portions of the product using the “Album of photographs and dishes” [26]. People taking part in the study wrote down the time, place, and exact composition of the meal on a specially prepared form. Then, the obtained information was entered into the computer program Diet 6.0 for planning and ongoing evaluation of individual and collective nutrition [27,28,29] and into the database, enabling the calculation of the consumption of polyphenolic compounds [30] and pantothenic acid [31]. The Diet 6.0 program is a rich database containing information about 101 food ingredients found in food products of various food groups. It is intended for planning menus and assessing individual and group nutrition. Additionally, it is used to calculate the nutritional value, which includes data on the content of protein, amino acids, fat and individual fatty acids, carbohydrates, fiber, cholesterol, minerals, and vitamins. Moreover, it is used for ongoing analysis of the consumption of individual food ingredients in the studied population groups compared to nutritional standards due to gender, age, and physiological condition [29]. The collected results were related to nutrition standards for men and women aged 19–30 [32] and for athletes [2,33]. The planned research received a positive opinion from the Bioethics Committee of the Nicolaus Copernicus University in Toruń (KB 250/2022).

### 2.4. Determination of the Antioxidant Activity of Blood Serum Using the DPPH• Radical

In order to determine the antioxidant activity of blood serum, the method of Chrzczanowicz et al. [34] with minor modifications was used. Before the experiment, a 100 µM DPPH• (2,2-diphenyl-1-pikrylohydrazyl) solution (Sigma Aldrich, St. Louis, NY, USA) was prepared by dissolving 4 mg of the reference substance in 100 mL of methanol (POCH, Warsaw, Poland). The 0.5 mL of the prepared DPPH• solution was added to Eppendorf tubes, 125 µL of the samples were added, vortexed, and then incubated for 60 min in the dark at room temperature. Absorbance was measured at 517 nm against a methanol reference. The control sample was a 100 µM methanol solution of DPPH• whose absorbance was measured at the beginning and at the end of the experiment. Five solutions of Trolox (TE, (+ −)-hydroxy-2,5,7,8-tetramethyl-chroman-2-carboxylic acid, Sigma Aldrich, St. Louis, NY, USA) with concentrations of 1.25–8.00 mg/L were prepared. The standard curve shows the dependence of the absorbance value of Trolox on its concentration. The results obtained were expressed in terms of Trolox equivalent (mg TE/100mL). The prepared standard curve was characterized by high linearity, which was confirmed by the high value of the correlation coefficient R2 = 0.939 (y = −0.0583x + 0.4491). In addition, the percentage ability of human milk to reduce the DPPH• radical was calculated:

% inhibition = ((A − Ab)/A) × 100

A—control sample absorbance.

Ab—mean value of absorbance of the tested serum.

### 2.5. Determination of the Ability of Blood Serum to Reduce Fe (III) Ions

The experiment used the FRAP method (ferric reducing antioxidant power) according to Benzie and Strain [35] with modifications. Before the experiment, 300 mM acetate buffer, pH 3.6 (a), 10 mM TPTZ (2,3,5-Triphenyl-tetrazolium chloride) in 40 mM HCl (b) and 20 mM FeCl_3_·6H_2_O (c) was prepared. The first stage of the assay was the preparation of the FRAP reagent by combining solutions a, b, and c in a ratio of 10:1:1. A blank was prepared, which was the FRAP reagent. The next stage was the application of 50 µL of the sample and 1.5 mL of FRAP reagent, vortexing, and then immediate reading of the absorbance of the sample (in 0 min.) at a wavelength of 593 nm and incubation at 37 °C for 4 min. After the incubation period, the samples were thoroughly mixed, and the absorbance at the same wavelength was measured again. Six solutions of ascorbic acid (Chempur, Warsaw, Poland) with concentrations of 100–1000 µM were prepared in order to determine the ability of blood serum to reduce Fe(III) ions. FRAP concentrations are expressed as ascorbic acid equivalent (µM). The correlation coefficient R2 = 0.999 (y = −0.0583x + 0.4491). The FRAP value was calculated from the following formula (ascorbic acid has a constant stoichiometric ratio of 2.0 in the FRAP test

(1) $$\mathrm{FRAP}=\frac{\mathrm{sample}\;\mathrm{absorbance}\;\mathrm{change}\;\mathrm{from}\;0\min\;\mathrm{to}\;4\;\min}{\mathrm{standard}\;\mathrm{absorbance}\;\mathrm{change}\;\mathrm{from}\;0\min\;\mathrm{to}\;4\;\min}\times C_{STD}\times 2\;[µM]$$

*C_STD_*—concentration of the standard solution

### 2.6. Determination of the Total Amount of Polyphenols in the Blood Serum

The method of Vazquez et al. [36] with modifications was used for the determination. The first stage of the assay was deproteinization of samples by mixing 0.1 mL of blood serum with 0.2 mL of 50% methanol, 10 µL of Carrez I and Carrez II reagents and 0.1 mL of acetonitrile. The samples were then thoroughly mixed and made up to 0.5 mL with 50% methanol. The samples were incubated at room temperature for 25 min, and after the incubation time had elapsed, they were centrifuged at 4500× *g* at 4 °C for 15 min.

The total sum of polyphenols was determined in the sample supernatant. An amount of 100 µL of the methanol extract was mixed with 1 mL of the previously prepared Folin–Ciocâlteu (FC) reagent (FC: H_2_O; 1:1, *v*/*v*) and with 3 mL of 20% Na_2_CO_3_. After vortexing, the mixture was incubated for 30 min in the dark at room temperature, then the absorbance was measured at 765 nm using a UV/VIS spectrophotometer (Biosense, Warsaw, Poland). The reference sample was 100 µL of methanol. Six water standard solutions of gallic acid (GAE) with concentrations of 0.01–0.10 mg/mL were prepared. The total content of polyphenols was calculated on the basis of a calibration curve describing the dependence of the absorbance value on the concentration of gallic acid (R2 = 0.998; y = 3.0842x − 0.0107). The results are presented as gallic acid equivalent in 1000 mL of blood serum (mg GAE/L).

### 2.7. Determination of the Biochemical Parameters

Parameters such as total protein, albumin, triglycerides, cholesterol, uric acid, glucose, ALAT (alanine aminotransferase), and ASAT (aspartate aminotransferase) were determined in blood serum using commercial, colorimetric tests from BioMaxima (Lublin, Poland) according to the attached procedure. Each sample was tested in triplicate.

### 2.8. Determination of the Micro- and Macronutrient

The concentrations of magnesium, calcium, chloride, phosphorus, and iron in blood serum were determined using commercial, colorimetric tests from BioMaxima (Lublin, Poland) according to the attached procedure. Each sample was assayed in triplicate.

### 2.9. Statistical Analysis

The results obtained during this research were subjected to statistical analysis using the Statistica ver. 13 program (StatSoft, Tulsa, OK, USA). The normality of the schedule was verified by the Shapiro–Wilk test. No normality of the distribution of the analyzed quantitative variables was found. The *p*-value 0 < 0.05 was considered significant. Differences between parameter values were calculated using a non-parametric test (Mann–Whitney U test). The variability of the parameters is presented in the form of a median, first quartile and third quartile. We also used the Spearman correlation coefficient to determine relationships between the relevant variables. In order to analyze many factors per variable in the studied groups, multiple linear regression was used. The variability of the parameters is presented in the form of a regression coefficient (B), error of the standard regression coefficient (SE), and the level of statistical significance *p* < 0.05.

## 3. Results

The control group included 7 men and 15 women aged 21 to 30 years. The average age is 24 ± 2. Due to the fact that people from the control group declared that they did not practice any sport, they were classified into the group of low physical activity. Based on the conducted research, it was found that 14% of people from the control group were overweight, and the remaining 86% had a normal weight. Statistically significant differences were found between the BMI of the study group and the control group (*p* = 0.001) (Table 1).

### 3.1. Daily Intake of Nutrients—24 h Interview

The study assessed 24 h intake of protein, fat, carbohydrates, fiber, cholesterol, glucose, and water, as well as energy requirements in athletes and in the control group. No statistically significant differences were observed between the groups in the analyzed parameters such as energy requirements, carbohydrates, fat, fiber, and cholesterol.

A higher intake of protein by 39.9% (*p* = 0.003), glucose by 7.7% (*p* = 0.044), and water by 31.5% (*p* = 0.002) was observed in the middle-distance runners compared to the control group (Table 2).

### 3.2. Daily Intake of Micro- and Macronutrients—24 h Interview

The study included 24 h intake of electrolytes (sodium, potassium), micronutrients (iron, copper, zinc, manganese) and macronutrients (calcium, phosphorus, magnesium) in the group of middle-distance runners and in the control group. No statistically significant differences were observed in the intake of potassium, calcium, magnesium, iron, copper, or manganese between the study groups. The intake of sodium was 37.3% (*p* = 0.011), phosphorus 31.6% (*p* = 0.006), and zinc 29.7% (*p* = 0.044) higher in the runners compared to the control group (Table 3).

Table 4 presents the percentage of achievement of the norm for micro- and macronutrients, relating the results to the nutritional norms for the population and athletes.

Numerous correlations were observed between the consumed nutrients, micro- and macroelements in the study groups, which are presented in Tables S3 and S4 (Supplementary Material).

### 3.3. Daily Intake of Fat-Soluble Vitamins—24 h Interview

The study included the intake of vitamin A, retinol, β-carotene, and vitamin E by middle-distance runners as well as by people from the control group. No significant statistical differences were found between the consumption of these vitamins in the control and study group, as shown in Table 5. The intake of vitamin A, β-carotene and vitamin E in the runners was higher than in the control group by 21%, 25% and 26%, respectively. Both in the study and control group, higher intake of vitamin A, retinol and vitamin E was found in men compared to women. The exception is the intake of β-carotene—women from the study group had a higher intake than men by about 55% (*p* = 0.474).

The range of vitamin A intake in the study and control groups was 359.68–2837.16 and 267.91–1950.87 µg/24 h, respectively. Whereas for retinol intake, the range was 113.62–803.29 and 80.77–1028.76 µg/24 h for the test and control groups. However, the highest intake values were recorded for β-carotene. β-carotene intake in both groups was 663.17–18,667.44 µg/24 h and 408.57–9437.35 µg/24 h. In turn, vitamin E intake was similar in both groups. The range in the study and control groups was 4.54–50.11 and 3.59–69.62 µg/24 h.

In the course of research, an analysis of correlations between all parameters was carried out. Correlation of Spearman’s rank order analysis showed a positive correlation between the intake of vitamin A by middle-distance runners and retinol (r = 0.60; *p* = 0.003) and vitamin E (r = 0.64; *p* = 0.001). All correlations are presented in the Supplementary Material (Table S1). Similar results were obtained in the control group. A high, positive correlation was found between the intake of vitamin A and β-carotene (r = 0.88; *p* = 0.001), where in the group of athletes this correlation was r = 0.81 (*p* = 0.001). The correlation results in the control group are presented in Table S2. It should be noted, however, that in the group of female middle-distance runners, the intake of vitamin A correlated with the intake of β-carotene at the level of r = 0.93 (*p* = 0.001). While in men, this correlation was r = 0.69 (*p* = 0.019). In turn, in the control group, a significant positive correlation was found in men and women, amounting to r = 0.86 and r = 0.84 (*p* = 0.001), respectively.

It is noteworthy that the study found that the consumption of β-carotene and vitamin A in middle-distance runners increased significantly with the increase in the number of meals per day (r = 0.52 and r = 0.48; *p* < 0.013). There was no significant correlation between these parameters in training women, but in men, such a correlation was found between the intake of vitamin E and the number of meals during the day (r = 0.68; *p* = 0.021).

Table 6 shows the percentage of achievement of the standard of fat-soluble vitamins, relating the results to nutritional standards for the population and for athletes. Only the intake of vitamin E in the control group and vitamin A in the study group exceeded the norms.

### 3.4. Daily Intake of Water-Soluble Vitamins—24 h Interview

Studies assessed the consumption by middle-distance runners of water-soluble vitamins: vitamin C, thiamine, riboflavin, niacin, folates, vitamin B6 and pantothenic acid. All results are presented in Table 7. The daily intake of water-soluble vitamins is about two times higher in the group of athletes than in the control group. Statistically significantly higher consumption of thiamine, riboflavin and pantothenic acid was noted. In the course of the research, it was found that men consumed more thiamine, riboflavin, niacin and vitamin B6 than women. Only for vitamin C and folates, higher daily intakes were found for women than for men. No statistically significant differences were found between the consumption of water-soluble vitamins for men and women of the study and control groups. The range of daily vitamin C intake for the group of middle-distance runners is 38.38–1212.11 mg/24 h and for the control group 25.09–564.81 mg/24 h. In turn, the range of thiamine 24 h intake in the study group is from 0.86 to 20.64 and in the control group from 0.62 to 8.92 mg. However, for riboflavin, it is 1.02–23.62 mg in the study group and 0.73–9.21 mg in the control group. Two times lower values were recorded for folate 24 h intake, where the range in the group of athletes was 202.137–891.42 µg, and in the control group 130.53–471.61 µg. Similar values were obtained for pantothenic acid. In the study group the range was 5.21–83.30 mg and in the control group 2.98–50.47mg. Moreover, the intake of niacin and vitamin B6 was about two times higher in the control group than in the study group. The range for niacin in the study group was 11.90–63.42 mg but in the control group 11.75–127.24 mg and for vitamin B6 1.04–19.77 mg (study group) and 0.92–41.37 (control group).

Positive correlations were found for serum parameters of middle-distance runners, such as intake of water-soluble vitamins listed in Table S2. The results of the Spearman’s rank correlation are presented in Table S1. It should be noted that in the case of training women, a high positive correlation was found between the consumption of riboflavin and thiamine (r = 0.99; *p* = 0.001), riboflavin and vitamin B6 (r = 0.92; *p* = 0.001) and vitamin B6 and folate (r = 0.96; *p* = 0.001). Whereas in the group of men, for the same parameters, it was noted that as thiamine intake increased, riboflavin intake (r = 0.79; *p* = 0.004) increased. In female runners, a significant positive correlation was observed between the consumption of pantothenic acid and niacin (r = 0.80; *p* = 0.003).

The obtained results were compared with the dietary intake norm for the population and athletes (Table 8). In the case of runners, the folate intake standards have not been met. In turn, in non-training people, only the intake of riboflavin and vitamin B6 was within the norm.

### 3.5. Daily Intake of Polyphenolic Compounds—24 h Interview

The study assessed the daily intake of polyphenols over three consecutive days. Table 9 presents the median together with the lower and upper quartiles of the obtained results. In the study, both women and men from the control group consumed more polyphenols compared to the group of runners (*p* = 0.016). The consumption of phenols was almost twice as low in middle-distance runners as in non-training people. The conducted research shows that the range of the average daily intake of phenols in the group of middle-distance runners is 11.49–1019.22 mg, and in the control group it is 28.75–37,971.42 mg. There were no statistically significant differences between the consumption of phenolic compounds by men and women in the group of middle-distance runners and the control group. It was noticed that training women consumed almost twice the amount of phenolic compounds compared to male runners. However, it should be noted that in the case of both women and men in both groups, the average number of meals was the same (n = 4).

### 3.6. Biochemical Parameters

To assess biochemical parameters from the runners’ blood serum, colorimetric tests were used to determine total protein, albumin, cholesterol, triglycerides, glucose, uric acid, ALAT, and ASAT (Table 10). A 24.6% higher uric acid concentration (*p* = 0.025) was observed in the runners’ group compared to the control group.

### 3.7. Micro and Macronutrients

Serum chloride concentration was 18.6% lower (*p* = 0.011) in middle-distance runners compared to the control group (Table 11). No significant differences were observed in calcium, magnesium, phosphorus, and iron concentrations between the study groups.

Correlations were observed between the tested biochemical parameters and the consumed ingredients, which are presented in Tables S5 and S6 (Supplementary Material).

### 3.8. Antioxidant Status

To assess the antioxidant properties of the serum of middle-distance runners and people from the control group, the FRAP method was used, which consists of determining the ability to reduce Fe^3+^ and DPPH· ions, which consists of reducing the 2,2-diphenyl-1- radical of picrylhydrazyl by antioxidant compounds contained in the serum. Moreover, the total content of phenolic compounds in blood serum was examined. All the results are presented in Table 12. The obtained results show that serum of athletes was characterized by a similar radical scavenging activity compared to the serum of the control group. In this study, the percentage of DPPH inhibition in the serum of middle-distance runners was found to be higher than in the control group by only approximately 8% (*p* = 0.238). Despite the fact that both the FRAP and DPPH methods are based on single electron transfer (SET), the present study showed that the Fe^3+^ ion reducing capacity of the serum of middle-distance runners is lower by about 8% compared to the serum of the control group (*p* = 0.369). In the course of research, it was found that the concentration of polyphenols in the blood serum in the group of runners is higher by 32% compared to the control group (*p* = 0.022).

The range of DPPH inhibition [%] in the study and control groups was 2.74–67.56% and 26.41–54.58%, respectively. In turn, the range of FRAP values was 151.33–875.09 µM for the study group and 121.12–796.34 µM for the control group, while the range of total polyphenol content in the serum of middle-distance runners was 16.83–105.54 mg/L, and in the control group, 42.50–106.14 mg/L.

During the study, a positive correlation was observed between the concentration of phenolic compounds in the serum of athletes and intake of β-carotene (r = 0.49; *p* = 0.029) and vitamin A (r = 0.43; *p* = 0.045), which is presented in Table S1. Moreover, a high correlation was found between the consumption of β-carotene and TPC in the serum of training men (r = 0.64; *p* = 0.035). It should be noted that in the case of training men, a positive significant correlation was found between the consumption of phenols and total antioxidant status determined by the FRAP [µM] (r = 0.78; *p* < 0. 004). What is more, it should also be emphasized that there is a negative correlation between the intake of niacin and the total antioxidant status [FRAP; µM] in female runners (r = −0.53; *p* = 0.096).

### 3.9. Multiple Regression

In order to examine the influence of additional factors (BMI, height, body weight, supplementation, gender) on the studied parameters, multiple regression was performed.

It was shown that in the control group, the age (B = −0.583, SE = 0.171, *p* = 0.004) of the participant influenced the concentration of chloride in the blood serum, the gender (B = 0.746, SE = 0.315, *p* = 0.030), age (B = −0.487, SE = 0.184, *p* = 0.017) and body weight (B = −0.732, SE = 0.296, *p* = 0.026) influenced the concentration of phosphorus, and the gender (B = 0.948, SE = 0.334, *p* = 0.012) influenced the concentration of uric acid. No statistically significant regressions were observed in the runners group.

## 4. Discussion

In this study, we focused our attention on examining the diet of middle-distance runners. We used information gathered through a 24 h interview, and we examined the relationship between an intake of nutrients and biochemical parameters, and the antioxidant status of blood serum.

Our study showed that the group of middle-distance runners consumed higher amounts of glucose (*p* = 0.044), protein (*p* = 0.003), and water (*p* = 0.002) compared to the control group. According to recommendations, elite runners should consume 40–70 kcal/kg/day of energy, with carbohydrates ranging from 250 to 1200 g/day and protein from 60 to 300 g/day [2]. Polish runners weighing between 55 and 71 kg provide their bodies with an adequate amount of energy (1762.34–2128.79 kcal), carbohydrates (245.37–284.00 g), and protein (89.50–110.64 g). However, runners with lower body weight could increase their carbohydrate intake, which is slightly below the recommended level. Hassapidou and Manstrantoni analyzing the diets of Greek middle-distance runners, reported lower values compared to our findings (energy: 1816 ± 549 kcal, protein: 65 ± 13 g, carbohydrates: 218 ± 77 g) [37]. In contrast, Japanese runners had greater nutritional requirements (3437 ± 505 kcal, 131 ± 33 g of protein, and 382 ± 19 g of carbohydrates) compared to Polish and Greek runners [38].

Energy expenditure during intense training or competition exceeds the previously mentioned levels. The energy requirements for cyclists weighing 60–80 kg competing in the Tour de France are estimated at 150–200 kcal/kg/day [39]. Athletes undergoing intensive training (1–2 sessions lasting 3–6 h, 5–6 times per week) need to consume carbohydrates at a level of 8–10 g/kg/day to maintain muscle glycogen stores [40]. Adequate energy intake, appropriate protein quantity and quality, as well as their combination with carbohydrates, are particularly important for maintaining lean body mass, supporting training adaptations, and optimizing performance [41].

One of the most effective ways to maintain exercise performance is proper hydration. Physical performance may be impaired with as little as a 2% loss of body mass due to sweating. Furthermore, a body mass loss exceeding 4% during physical activity may lead to heat-related illness [42]. To maintain fluid and electrolyte balance, athletes are advised to consume 0.5–2 L of fluids during exercise [2]. Casa et al. recommend a total daily fluid intake of 2–4 L [43]. Our results indicate that Polish middle-distance runners aged 55–71 years consume between 3176.80 and 4378.87 g of water per day. Jimenez-Alfagemez et al., in a study on hydration and nutrition among marathon runners, observed that fluid intake was at the lower end of the recommended range [44].

Minerals are essential for numerous metabolic processes, and sudden changes in sodium, potassium, and magnesium levels are often observed during physical activity. Deficiencies in vitamins or minerals can impair exercise performance [1]. Our study found that middle-distance runners consumed higher amounts of sodium (*p* = 0.011), phosphorus (*p* = 0.006), and zinc (*p* = 0.019) compared to the control group, exceeding the recommended intake for these minerals. Conversely, the non-training group showed insufficient dietary intake of zinc, magnesium, calcium, and potassium relative to recommended values. Previous studies show similar patterns across populations. Sugiura and Kobayashi reported that Japanese middle-distance runners consumed 880 ± 261 mg of calcium and 20 ± 5 mg of iron, reflecting higher iron but lower calcium intake compared to our participants [38]. Nuviela et al. found that Spanish female runners consumed 259 ± 69.5 mg magnesium, 9.2 ± 2.2 mg zinc, and 2.29 ± 2.14 mg copper, indicating higher magnesium and zinc but lower copper intake relative to our results [45]. Alves et al. observed comparable microelement intake to that of our participants [46].

Additionally, we observed that the serum of middle-distance runners was characterized by lower chloride levels (*p* = 0.011) and higher uric acid levels (*p* = 0.025) compared to the control group.

Chloride concentration in the human body is tightly regulated by the kidneys. Physiological changes in serum chloride concentration are rare and may be caused by factors such as intense physical exercise, pregnancy, and dehydration/overhydration [47,48,49,50]. Dysregulated chloride levels have been associated with metabolic acidosis, impaired organ function, and disruptions in hemodynamic stability [50]. Intense physical activity affects fluid and electrolyte balance due to the loss of electrolytes through sweat. Choi et al. reported that chloride concentration in sweat increased from 12.0 ± 5.9 mM to 31.4 ± 16 mM following exercise, which may lead to decreased serum chloride levels in runners [47]. Similarly, Cohen and Zimmerman also observed a reduction in serum chloride in runners [48].

Uric acid is believed to be a scavenger of free radicals, superoxide, and hydrogen peroxide. Maintaining normal uric acid levels provides antioxidant and neuroprotective effects [51]. High uric acid levels (above 6.8 mg/dL) can cause pro-inflammatory responses, increase antioxidant stress, reduce renal blood flow, and impair kidney function [52]. The increase in serum uric acid in middle-distance runners results from intensified purine metabolism during anaerobic exercise. Intense training leads to ATP breakdown and inhibition of uric acid excretion due to elevated lactate, which can result in its accumulation. Elevated uric acid levels have also been reported bySjödin and Hellsten Westing [53] and Devrilmez and Soslu [54].

One of the main tasks of vitamin E is protection against the formation of reactive oxygen species (ROS) and exhibiting anti-inflammatory properties [13,55]. Elite athletes are exposed to training loads that can trigger overtraining syndrome (OTS), a phenomenon in which there is an increase of pro-inflammatory markers and, consequently, a decrease in training efficiency [2,56]. Therefore, it is extremely important to control the amount of vitamin E in the daily food ration of middle-distance runners, as vitamin E can inhibit the secretion of inflammatory-mediating molecules, such as eicosanoids and the enzyme cyclooxygenase-2 (COX-2) [55]. Our own research showed a deficiency [2] of vitamin E in middle-distance runners at the level of 71% for women and 88% for men (Table 3), which may contribute not only to reduced antioxidant protection, but also to the inhibition of inflammation and muscle pain during the recovery period after exhausting training and endurance exercises [57]. On the other hand, in the studies of Passos et al., a daily intake of vitamin E of 14.0 ± 1.5 mg/day was observed in marathon runners, close to the norm for athletes [58]. Similar results were obtained by Jeoung & Kim in studies conducted on 21 disabled Korean athletes (14.57 mg/day) [59]. In turn, research conducted among cyclists (n = 31) showed that the daily intake of vitamin E was 10 ± 1.0 mg [60]. There is evidence that training at high altitudes may require adequate or increased intake of vitamin E to improve exercise performance [2]. Furthermore, it should be mentioned that about 20–60% of tocopherol is absorbed from the gastrointestinal tract. Moreover, as is the case with other fat-soluble vitamins, the absorption of vitamin E depends on the body fat content, bile salts, and pancreatic enzymes [61]. Previous research indicates that college rowers who used a low-fat versus a high-fat diet consumed significantly less vitamin E (2.9 mg of vitamin E/day) than those in the high-fat group (9.8 mg of vitamin E/day). Therefore, when assessing the intake of fat-soluble vitamins, it is also advisable to pay attention to dietary fat intake. Impaired absorption of vitamin E as a result of a low-fat diet may result in insufficient levels of vitamin E [13]. Therefore, it is extremely important that the intake of vitamin E (expressed as α-tocopherol) meets the recommended daily allowance. Moreover, according to Santos et al. [62], Vitamin E supplementation (250 mg) 1 h before exercise reduces markers of post-exercise cell damage, hypoxia, and reduces the concentration of inflammatory cytokines, which may suggest a potential protective effect against exercise-induced inflammation. However, during intense exercise, free radical or reactive oxygen and nitrogen species (RONS) production increases and can inhibit muscle contractile function, leading to muscle fatigue and decreased performance [13]. Considering the role of antioxidants in protection against free radicals, it has become common practice among athletes to consume antioxidant supplements to fight muscle damage and fatigue and increase performance. Evidence suggests that antioxidant supplementation may interfere with the adaptation of exercise training. Therefore, it is even more important to control the level of natural antioxidant intake through diet. Reactive species formed during exercise may improve aerobic capacity and muscle hypertrophy, by stimulating molecular pathways via proteins, including those activated by peroxisome proliferators receptor c coactivator (PGC1-α) and mitogen-activated protein kinases (MAPKs) [13]. As it turns out, the intake of vitamin E (400 IU/day) and vitamin C (1000 mg/day) prevents the induction of PGC1-α and mitochondrial biogenesis and is also a key endogenous antioxidant enzyme in human skeletal muscle. However, it should be noted that the indicated intake values of vitamin E and C are only possible to obtain through additional supplementation. Our own research was an attempt to determine the correlation between daily intake of vitamin E and other water- and fat-soluble vitamins. There was a significant relationship between the intake of retinol and vitamin A and all other water-soluble vitamins that were included in the study (vitamin C, thiamine, riboflavin, niacin, folates, vitamin B6 and pantothenic acid). However, it should be emphasized that only in the case of vitamin E and folate, the daily intake was not reached [2]. In the case of folates, the percentage of meeting the norm for middle-distance runners was 94% and for runners, 89%. A Brazilian study involving 44 amateur runners (men) found that folic acid intake was 275 ± 32 mg per day [58]. Therefore, the implementation of reference daily values was approximately 69% [2,58]. Higher than the recommended norms (400 µg/day), the average intake of folic acid was recorded by Korean researchers in disabled athletes, including two track and field athletes, who were found to have an average of 413.65 ug/day [59].

The results obtained in our own research among middle-distance runners were similar to those obtained by Brazilian researchers among marathon runners [58]. The median vitamin A intake among marathon runners was 1061.0 µg, and the median intake among middle-distance runners was 1067.4 µg. In our research, for the women-only control group, the total three-day average vitamin A intake did not exceed the recommended daily allowance (RDA) of 700 µg (89%). However, among Spanish cyclists (n = 31), the median consumption of vitamin A was 2539.0 µg/24 h [60]. Nutritional deficiencies of vitamin A are particularly important as it plays a key role in determining the differentiation of B lymphocytes into IgA-secreting cells present in the intestinal mucosa. The reduced content of vitamin A reduces the production of IgA, causing malabsorption and changes in the deposition of adipose tissue [57]. Research indicates that aerobic exercise is the main cause of increased levels of free oxygen radicals (e.g., superoxide radicals O2•, hydroxyl radicals OH•; HO_2_ hydroperoxide radicals•; and lipid peroxide radicals LOO•) [63]. According to Moussa et al., vitamin A removes lipid peroxide radicals [64]. By removing the superoxide radical via an addition reaction to the retinol β-ion ring, the resulting tertiary and highly conjugated trans-retinol carbon radical intermediate is considered relatively stable, and its reactivity is not sufficient to induce further lipid peroxidation on its own.

Although vitamin C was consumed by middle-distance runners in accordance with recommendations (median), the percentage of people with insufficient vitamin C intake, below 75 mg/day, was as much as 32%. However, it should be noted that vitamin C intake varied among people practicing different sports. The average intake among marathon runners was 144.0 mg [58] and among cyclists 133.0 mg [60]. On the other hand, in middle-distance runners, this intake was lower (106.29 mg). Researchers’ opinions on vitamin C supplementation are divided. Some studies indicate positive results related to the reduction in markers of muscle damage after intense exercise with some form of vitamin C supplementation. However, it should be emphasized that the authors of the literature review indicate that due to the possibility of weakening physiological adaptation to training, long-term supplementation with large doses of vitamin C is not recommended [65]. Therefore, it is recommended that athletes obtain antioxidants through a nutrient-rich diet rather than through the use of supplements. Additionally, studies by other authors indicate that supplementation with vitamins C and E alleviates the adoption of skeletal muscles, which was demonstrated by the activity of superoxide dismutase and mitochondrial transcription factor A [13]. Bioactivity and metabolism of antioxidant compounds in the daily food ration are different and have a significant impact on antioxidant properties. An example is vitamin E, which may be a pro-oxidant, not an antioxidant. This is observed in vitro at depletion of other antioxidant compounds (ascorbates) that are necessary for the reduction in vitamin E [66]. This results in the accumulation of an oxidized form of vitamin E, which oxidizes lipids. It is also particularly important that a daily food ration rich in antioxidants (food combinations) may counteract the absorption and protective effect of flavonoids.

Polyphenols are a group of over 8000 chemical compounds with various structures that have antioxidant and anti-inflammatory effects. Their main source is the above-ground parts of plants, which give plants color and provide protection against UV radiation. It is recommended for athletes to consume approximately 1000–2000 mg/day [33]. The median consumption of phenolic compounds in Poland is approximately 1000 mg [67]. The median intake of polyphenolic compounds was higher than the recommended norms in the control group than in middle-distance runners. Despite the increased demand for phenolic compounds in the face of oxidative stress in the body, the daily food ration of people practicing middle-distance running was poor. It should be noted, however, that our own results indicate that only one athlete (5%) consumed an average of 1019.22 mg/day in his daily food ration. The remaining athletes consumed less than 627.72 mg/day of phenolic compounds. It should be noted, however, that the databases available for calculating the daily intake of phenolic compounds are not rich enough to include all dietary sources of phenols. Therefore, there is a strong need to expand the database of computer programs for dietary and nutritional purposes. At this point, attention should be paid to the bioavailability of phenols, which depends, among other things, on the degree of their release from the food matrix, absorption from the gastrointestinal tract and pre-systemic metabolism [68]. An extensive review article indicates the role of individual flavonoids or phenolic acids in the exercise of athletes [68]. Isoflavone intake at a dose of 70 mg/day is effective in reducing fat mass, plasma γ-glutamyltransferase levels and CRP levels combined with 6 months of exercise. Reducing the amount of pro-inflammatory markers in plasma and calf muscles is owed to catechin [69]. In our research, water infusion of Camelia sinensis was occasionally included in the daily food ration of the study group. While in the control group, the consumption of coffee and aqueous infusion from Camelia sinensis or green tea was very frequent, which translated into a statistically significantly higher consumption of phenols compared to the study group (*p* = 0.016). According to a review article [68], green tea and Camelia sinensis infusions contribute to reducing the number of markers of muscle damage after a series of exercises. Consumption of flavonoids, phenolic acids and isoflavones present in raw materials of plant origin contributes to increasing the antioxidant capacity of serum [70]. However, in our own research, we did not find statistically significant correlations between the consumption of antioxidant compounds and the antioxidant status of blood serum. As other researchers point out, a significant increase in the antioxidant capacity of serum, measured in the ABTS (2,2′-azino-bis (3-ethylbenzthiazoline-6-sulphonic acid) and FRAP tests, is noticeable 1 h after consuming Golden Delicious apples (4.2% and 10.2%, respectively) and Catarina AJ (5.5% and 10.6%, respectively) compared to the baseline values [71].

Exposure to RONS (during intense exercise) causes skeletal muscles to adapt, enabling them to cope with subsequent stressors in the future. Such training adaptations include strengthening the body through increasing the production of endogenous antioxidants, which may provide sufficient protection to the body without having to increase the intake of exogenous antioxidants (e.g., dietary supplements) [13]. In turn, the study conducted by Koivisto et al., as reported by the authors themselves, is probably the first study aimed at assessing whether increased consumption of foods rich in antioxidants during a 3-week moderate altitude training camp affects systemic oxidative stress and inflammation at rest and in response to maximal exercise in elite endurance athletes [72]. It has been reported that it is possible to increase the plasma antioxidant capacity of competitive athletes during training by increasing the daily intake of typical antioxidant-rich foods. The research focused on the consumption of natural foods rich in antioxidants (cocoa, juices, fruits, nuts). By doubling the daily intake of antioxidant-rich foods, it was possible to attenuate the altitude-induced increase in micro-CRP, IL-13, and the strong trend in IL6 [72]. Daily food rations rich in antioxidants cause an increase in FRAP. The authors indicate that antioxidant capacity was higher in the antioxidant group compared to the control group. Eating antioxidant-rich foods results in a gradual increase in endogenous antioxidant levels [72].

The presented pilot studies showed that the intake of folates and vitamin E in athletes was too low. Moreover, the obtained results clearly indicate that the consumption of polyphenolic compounds found in the above-ground parts of plants should be increased. Neglecting eating habits in people undergoing intense physical exercise may result in serious health consequences.

This study is a preliminary description of the intake of macronutrients, micronutrients, water, and fat-soluble vitamins by middle-distance runners. This is the first study to analyze the nutrition of Polish middle-distance runners and to investigate the relationship between nutrition and biochemical parameters and the antioxidant status of serum from the runners.

The limitations of our study include the small number of study participants and the limited number of biochemical parameters determined in the serum from the runners. The narrow study group results from the selection of a specific region of Poland (Kuyavian-Pomeranian Voivodeship); therefore, in the future it is worth expanding the study to include participants from other regions of Poland. An additional limitation of the study is the use of a 3-day dietary interview to assess the participants’ nutritional status. It is worthwhile to expand the interview to a minimum 7-day dietary interview in the future. Additionally, it is worth noting that the control group did not take supplements, compared to the study group. This factor may also influence changes in the biochemical parameters of blood serum. Using multiple regression in statistical analyses, we did not demonstrate an effect of supplementation on the parameters studied, but this is worth considering in future studies. Gender may be an additional factor influencing the results. Due to the small sample size, statistical comparisons between gender groups were not performed. Multiple regression revealed that this factor influenced uric acid concentration in the control group. No other correlations were observed. Future research should be expanded to include gender as a factor influencing the parameters studied. A final limitation of this study is that samples were collected after training, from individuals who were not fasting. These factors may have influenced the analyzed parameters.

However, it should be clearly emphasized that these results increase knowledge about the body’s need for antioxidants, such as vitamin A, beta-carotene, vitamin C and polyphenols.

## 5. Conclusions

The diet of middle-distance runners is well-balanced, but in some aspects requires some modification. In particular, attention should be paid to supplementing folate and vitamin E deficiencies in the diet. Additionally, middle-distance runners are at risk of deficient dietary intake of phenols. Therefore, when composing daily menus, runners should pay special attention to increasing the consumption of olive oil, almonds or hazelnuts, which could contribute to the increase of consumption of not only vitamin E, but also polyphenols. The presented research requires extension to a larger number of subjects, considering dietary preferences resulting in use of various diets, including elimination ones.

## Figures and Tables

**Table 1:** Characteristics of the study groups.

	Control Group (Me; Q_25_–Q_75_)			Study Group (Me; Q_25_–Q_75_)			*p*
	W (n = 15)	M (n = 7)	Total (n = 22)	W (n = 11)	M (n = 11)	Total (n = 22)	
Body mass [kg]	60.0 (57.0–68.0)	82.0 (75.0–87.0)	65.15 (59.0–75.0)	55.0 (48.0–71.0)	68.0 (62.0–76.0)	63.50 (55.0–71.0)	0.391
Height [cm]	168.0 (164.0–170.0)	178.0 (176.50–183.0)	170.0 (167.0–178.0)	167.0 (163.0–172.0)	181.0 (171.0–184.0)	172.0 (167.0–181.0)	0.411
BMI [kg/m^2^]	22.0 (20.0–24.0)	25.0 (24.0–27.0)	23.0 (21.0–26.0)	19.0 (18.0–21.0)	20.0 (19.0–21.0)	20.0 (19.0–21.0)	**0.001**
Age [years]	24.0 (23.0–24.0)	23.0 (22.0–24.0)	23.0 (23.0–24.0)	24.0 (22.0–26.0)	24.0 (21.0–25.0)	24.0 (21.0–25.0)	0.720

Me—median; Q_25_—first quartile; Q_75_—third quartile; W—woman; M—man; BMI [kg/m2]: <18.5—underweight; 18.6–24.9—normal weight; 25.0–29.9—overweight; 30.0–34.9—class I obesity; 35.0–39.9—class II obesity; ≥40—class III obesity; n—numbers; *p*—level of statistical significance (Mann–Whitney U test between the study group and the total control group); bold—statistically significant result.

**Table 2:** Median daily intake of nutrient.

	Control Group (Me; Q_25_–Q_75_)			Study Group (Me; Q_25_–Q_75_)			*p*
	W (n = 15)	M (n = 7)	Total (n = 22)	W (n = 11)	M (n = 11)	Total (n = 22)	
Energy [kcal]	1967.62 1720.17–2616.70	2085.22 1355.92–2485.06	1993.10 1589.3–2485.06	2167.64 1860.90–2928.79	2108.22 1619.32–3547.74	2108.22 1762.34–2128.79	0.156
Water [g]	2770.08 2326.20–3827.90	2370.81 1665–3346.58	2720.04 2177.77–3703.93	3391.19 2916.40–4280.36	3949.75 3287.30–4396.30	3577.19 3176.80–4378.87	**0.002**
Protein [g]	80.26 50.04–98.38	85.44 63.06–95.26	80.55 57.38–95.29	113.53 96.29–142.38	89.98 78.94–149.54	112.69 89.50–140.64	**0.003**
Fat [g]	74.06 58.06–95.01	60.53 40.46–101.51	72.36 51.45–95.91	70.96 48.68–95.18	78.23 55.21–104.31	76.63 50.00–103.28	0.782
Carbohydrates [g]	261.19 174.49–310.14	253.36 199.63–299.69	257.28 199.63–308.13	311.51 282.70–347.34	306.82 223.32–491.28	306.82 245.37–284.00	0.069
Cholesterol [mg]	254.83 182.01–339.82	317.59 194.38–354.25	261.92 189.68–351.07	315.05 259.05–500.46	349.10 219.06–454.31	332.03 250.82–454.31	0.066
Glucose [g]	4.74 3.75–11.36	5.17 4.06–7.44	9.38 8.18–11.47	11.32 8.93–17.45	6.93 4.35–13.75	10.10 4.86–13.72	**0.044**
Fiber [g]	18.24 15.86–30.01	19.21 15.76–21.04	18.72 15.86–21.21	24.72 17.85–29.33	25.14 15.85–36.44	25.14 17.85–29.33	0.117

Me—median; Q_25_—first quartile; Q_75_—third quartile; n—number; *p*—level of statistical significance (Mann–Whitney U test between the study group and the total control group); bold—statistically significant result.

**Table 3:** Median daily intake of micro- and macronutrients.

	Control Group (Me; Q_25_–Q_75_)			Study Group (Me; Q_25_–Q_75_)			*p*
	W (n = 15)	M (n = 7)	Total (n = 22)	W (n = 11)	M (n = 11)	Total (n = 22)	
Sodium [mg]	2717.59 1960.86–3770.64	2432.72 1991.20–3472.28	2688.82 1991.19–3577.37	3723.04 3422.07–4462.90	3151.99 2713.75–4181.40	3690.80 3003.60–4181.40	**0.011**
Potassium [mg]	3455.95 2489.72–4935	2880.54 2619.25–3559.81	3155.74 2619.25–3795.75	3265.04 3090.31–3434.24	3297.17 2315.98–5105.81	3276.84 2490.27–3591.48	0.894
Calcium [mg]	813.01 483.14–1100.91	714.78 631.88–1086.93	792.27 549.57–1086.93	1073.33 677.81–1491.54	1024.44 620.86–1142.80	1024.94 677.80–1296.90	0.112
Phosphorus [mg]	1293.66 956.66–1630.55	1322.79 943.72–1413.25	1308.22 956.66–1486.18	1764.31 1440.54–2050.37	1532.29 1268.17–2271.64	1721.90 1414.23–2050.37	**0.006**
Magnesium [mg]	363.37 265.52–447.22	344.46 258.40–373.63	351.68 263.32–423.69	378.08 278.29–483.13	466.00 276.71–576.00	282.61 278.29–487.72	0.213
Iron [mg]	13.86 8.78–16.93	12.16 9.02–15.33	13.66 9.02–16.27	14.66 11.12–16.78	14.42 10.05–22.86	14.42 11.12–17.18	0.305
Zinc [mg]	9.43 6.71–14.90	8.97 8.20–10.48	9.38 8.18–11.47	12.05 10.15–14.40	12.57 9.50–16.46	12.17 10.15–14.40	**0.019**
Copper [mg]	1.39 0.94–2.16	1.21 0.93–1.43	1.33 0.94–1.70	1.35 1.25–1.67	1.64 0.87–2.31	1.37 1.09–1.91	0.555
Manganese [mg]	4.84 3.2–6.77	4.06 3.91–4.31	4.10 3.84–6.19	4.98 3.97–7.08	5.76 5.01–7.78	5.64 4.33–7.08	0.069

W—woman; M—man, Me—median; Q_25_—first quartile; Q_75_—third quartile; n—number; *p*—level of statistical significance (Mann–Whitney U test between the study group and the total control group); bold—statistically significant result.

**Table 4:** Percentage of nutrition standards for micro- and macronutrients.

		Nutritional Standard			Percentage of Standard Compliance		
**Control Group (n = 22)**							**Ref.**
		**Kind**	**W (n = 15)**	**M (n = 7)**	**W**	**M**	
Sodium	mg	RDA	1500	1500	181	162	[25]
		EAR	1500	1500	181	162	
Potassium	mg	RDA	3500	3500	99	82	
		EAR	3500	3500	99	82	
Calcium	mg	RDA	1000	1000	81	71	
		EAR	800	800	101	89	
Phosphorus	mg	RDA	700	700	184	189	
		EAR	580	580	223	228	
Magnesium	mg	RDA	310	400	117	86	
		EAR	255	330	142	104	
Iron	mg	RDA	18	10	78	120	
		EAR	8	6	175	200	
Zinc	mg	RDA	8	11	117	82	
		EAR	6.8	9.4	100	96	
**Study Group (n = 22)**							
			**W (n = 11)**	**M (n = 11)**	**W**	**M**	
Sodium	mg	RDA	1500	1500	248	210	[26]
Potassium	mg	RDA	2000	2000	163	165	
Calcium	mg	RDA	1000	1000	107	103	
Phosphorus	mg	RDA	700	700	252	219	
Magnesium	mg	RDA	320	420	118	111	
Iron	mg	RDA	18	8	80	131	
Zinc	mg	RDA	8	11	157	111	

RDA—Recommended Dietary Allowances, EAR—Estimated Average Requirement, W—woman, M—man.

**Table 5:** Median daily intake of fat-soluble vitamins.

	Control Group (Me; Q_25_–Q_75_)			Study Group (Me; Q_25_–Q_75_)			*p*
	W (n = 15)	M (n = 7)	Total (n = 22)	W (n = 11)	M (n = 11)	Total (n = 22)	
Vitamin A [µg]	652.50 (601.95–1208.02)	1071.07 (891.80–1777.15)	877.01 (601.94–1233.06)	914.62 (794.85–1424.47)	1098.10 (672.58–1641.47)	1067.40 (678.69–1424.47)	0.245
Retinol [µg]	357.01 (244.60–428.42)	417.85 (271.96–684.96)	357.01 (271.76–466.99)	299.66 (278.99–450.47)	392.61 (324.78–637.50)	361.82 (290.37–531.27)	0.699
β-Carotene [µg]	1836.58 (1408.78–4970.09)	4504.70 (1808.18–5536.97)	2911.67 (1468.20–4970.09)	4572.87 (2005.84–7326.15)	2933.86 (1337.45–6454.11)	3641.22 (2005.84–6454.11)	0.474
Vitamin E [µg]	9.09 (7.138–15.34)	13.88 (5.684–41.25)	10.08 (7.14–15.47)	10.69 (5.872–20.22)	13.24 (6.84–22.52)	12.77 (6.84–20.22)	0.504

Me—median; Q_25_—first quartile; Q_75_—third quartile; Vitamin E as eq. α-tocopherol; n—number; *p*—level of statistical significance (Mann–Whitney U test between the study group and the total control group).

**Table 6:** Percentage of nutrition standards for fat-soluble vitamins.

		Nutritional Standard			Percentage of Standard Compliance		
**Control Group (n = 22)**							**Ref**.
		**Kind**	**W (n = 15)**	**M (n = 7)**	**W**	**M**	
Vitamin A	µg	RDA	700	900	89	119	[25]
		EAR	500	630	131	170	
Vitamin E	µg	AI	8	10	114	139	
**Study Group (n = 22)**							
			**W (n = 11)**	**M (n = 11)**	**W**	**M**	
Vitamin A *	µg	RDA	700	900	130	122	[26]
Vitamin E **	µg	RDA	15	15	71	88	

RDA—Recommended Dietary Allowances, EAR—Estimated Average Requirement, AI—Adequate Intake, W—woman, M—man, *—mg retinol equivalent, **—mg tocopherol equivalent.

**Table 7:** Median daily intake of water-soluble vitamins.

	Control Group (Me; Q_25_–Q_75_)			Study Group (Me; Q_25_–Q_75_)			*p*
	W (n = 15)	M (n = 7)	Total (n = 22)	W (n = 11)	M (n = 11)	Total (n = 22)	
Vitamin C [mg]	77.34 (44.19–173.72)	75.66 (28.54–116.83)	76.50 (40.65–145.08)	125.53 (66.80–298.70)	94.40 (55.46–191.77)	106.29 (66.80–191.71)	0.148
Thiamine [mg]	0.93 (0.70–1.49)	1.09 (0.83–1.83)	0.99 (0.79–1.49)	1.33 (1.05–3.83)	2.08 (1.09–4.15)	1.48 (1.09–3.83)	**0.004**
Riboflavin [mg]	1.61 (1.21–1.91)	1.62 (1.17–2.29)	1.62 (1.21–1.93)	1.79 (1.54–4.15)	2.38 (1.71–5.90)	2.07 (1.58–4.15)	**0.014**
Niacin [mg]	17.13 (13.10–24.68)	25.66 (17.77–42.94)	18.20 (13.10–25.66)	23.77 (14.56–29.60)	29.52 (22.67–50.77)	27.35 (17.74–37.95)	0.084
Folates [µg]	304.24 (265.40–359.43)	337.15 (231.88–413.22)	319.60 (265.40–359.43)	374.11 (235.30–510.41)	357.28 (284.87–521.50)	365.70 (284.87–510.41)	0.080
Vitamin B6 [mg]	1.82 (1.54–3.58)	3.29 (1.45–7.77)	1.83 (1.46–3.58)	2.23 (1.61–3.12)	2.64 (1.93–4.38)	2.39 (1.88–3.95)	0.392
Pantothenic acid [mg]	3.98 (3.74–4.68)	4.80 (4.46–5.38)	4.33 (3.76–4.80)	6.60 (5.34–7.83)	6.90 (5.50–11.10)	6.78 (5.50–11.10)	**0.001**

Me—median; Q_25_—first quartile; Q_75_—third quartile; W—woman; M—man, n—number; *p*—level of statistical significance (Mann–Whitney U test between the study group and the total control group); bold—statistically significant result.

**Table 8:** Percentage of nutrition standards for water-soluble vitamins.

		**Nutritional Standard**			**Percentage of Standard Compliance**		
**Control Group (n = 22)**							**Ref.**
		**Kind**	**W (n = 15)**	**M (n = 7)**	**W**	**M**	
Vitamin C	mg	RDA	75	90	103	84	[26]
		EAR	60	75	129	101	
Thiamine	mg	RDA	1.10	1.30	85	84	
		EAR	0.90	1.10	103	99	
Riboflavin	mg	RDA	1.10	1.30	146	125	
		EAR	0.90	1.10	179	147	
Folates	µg	RDA	400	400	76	84	
		EAR	320	320	95	105	
Vitamin B6	mg	RDA	1.30	1.30	140	253	
		EAR	1.10	1.10	165	299	
Pantothenic acid	mg	AI	5.0	5.0	80	96	
**Study Group (n = 22)**							
			**W (n = 11)**	**M (n = 11)**	**W**	**M**	
Vitamin C	mg	RDA	75	90	167	104	[26]
Thiamine	mg	RDA	1.1	1.2	121	173	
Riboflavin	mg	RDA	1.7	1.3	105	183	
Folates	µg	RDA	400	400	94	89	
Vitamin B6	mg	RDA	1.3	1.3	171	203	
Pantothgenic acid	mg	RDA	5.0	5.0	138	136	

RDA—Recommended Dietary Allowances, EAR—Estimated Average Requirement, AI—Adequate Intake, W—woman, M—man.

**Table 9:** Median daily intake of phenols [mg].

Control Group (Me; Q_25_–Q_75_)			Study Group (Me; Q_25_–Q_75_)			*p*
W (n = 15)	M (n = 7)	Total (n = 22)	W (n = 11)	M (n = 11)	Total (n = 22)	
436.30 (230.31–505.84)	454.82 (230.58–1025.05)	445.56 (230.31–617.73)	313.13 (59.23–505.71)	166.44 (90.91–284.05)	255.03 (90.90–349.95)	**0.016**

Me—median; Q_25_—first quartile; Q_75_—third quartile; W—woman; M—man, n—number; *p*—level of statistical significance (Mann–Whitney U test between the study group and the total control group); bold—statistically significant result.

**Table 10:** Serum biochemical parameters.

	Control Group (Me; Q_25_–Q_75_)			Study Goup (Me; Q_25_–Q_75_)			*p*
	W (n = 15)	M (n = 7)	Total (n = 22)	W (n = 11)	M (n = 11)	Total (n = 22)	
Total protein [g/dL]	8.33 7.59–9.22	7.59 7.02–9.39	8.18 7.47–9.22	8.33 7.76–8.98	7.84 7.39–8.86	8.16 7.51–8.9	0.953
Albumin [g/dL]	5.04 4.61–5.49	5.28 4.84–6.02	5.09 4.60–5.50	5.37 4.22–6.04	5.22 4.97–5.44	5.29 4.97–5.78	0.463
Cholesterol [mg/dL]	157.26 141.7–168.29	166.05 141.7–184.3	159.13 141.70–171.50	178.78 152.77–214.76	188.16 146.68–214.7	161.55 158.71–214.7	0.056
Triglycerides [mg/dL]	103.67 87.16–123.6	99.5 89.9–148.6	100.46 87.16–134.86	113.76 92.66–145.87	96.33 87.16–121.10	97.29 89.91–137.61	0.789
Glucose [mg/dL]	66.12 58.54–78.16	66.72 63.84–71.90	66.12 61.81–79.15	71.32 59.88–80.98	69.54 64.93–81.70	70.43 64.93–80.98	0.229
Uric acid [mg/dL]	4.06 3.023–4.9	5.83 4.73–6.84	4.35 3.54–5.63	5.21 3.23–8.23	5.94 5.1–8.54	5.42 4.64–8.23	**0.025**
ALAT [U/L]	17.8 13.7–24.9	20.6 20.3–30.2	19.55 14.60–27.40	21 13.7–23.9	27.4 20.2–30.7	22.85 15.70–27.40	0.667
ASAT [U/L]	12 9–17	16 16–23	15.50 10–17	12.5 10–22.7	17 13–19	15.35 12–19	0.634

Me—median; Q_25_—first quartile; Q_75_—third quartile; W—woman; M—man, n—number; *p*—level of statistical significance (Mann–Whitney U test between the study group and the total control group); bold—statistically significant result.

**Table 11:** Serum micro- and macronutrients.

	Control Group (Me; Q_25_–Q_75_)			Study Goup (Me; Q_25_–Q_75_)			*p*
	W (n = 15)	M (n = 7)	Total (n = 22)	W (n = 11)	M (n = 11)	Total (n = 22)	
Chlorides [mmol/L]	91.77 88.14–120.34	118.16 110.41–130	110.65 88.86–128.33	88.62 84.99–91.53	95.88 89.35 96.70	90.07 87.17–96.37	**0.011**
Calcium [mg/dL]	14.74 9.37–18.42	11.16 8.32–17.89	12.95 9.37–17.89	9.47 9.05–10.84	9.47 8.42–12.84	9.47 8.84–12.53	0.449
Magnesium [mg/dL]	2.10 1.90–2.47	2.01 1.74–2.80	2.08 1.9–2.47	1.91 1.73–2.12	2.21 1.81–2.53	1.94 1.80–2.28	0.357
Phosphorus [mg/dL]	3.86 3.32–5.62	6.38 4.68–7.27	4.62 3.68–7.10	3.7 3.18–4.76	4.0 3.54–4.74	3.85 3.54–4.74	0.202
Iron [µg/dL]	95.9 75.41–142.62	82.79 68.03–116.38	93.85 75.41–124.59	130.33 72.13–146.72	99.18 92.79136.07	107.78 77.05–140.16	0.506

Me—median; Q_25_—first quartile; Q_75_—third quartile; W—woman; M—man, n—number; *p*—level of statistical significance (Mann–Whitney U test between the study group and the total control group); bold—statistically significant result.

**Table 12:** Serum antioxidant status.

	Control Group (Me; Q_25_–Q_75_)			Study Goup (Me; Q_25_–Q_75_)			*p*
	W (n = 15)	M (n = 7)	Total (n = 22)	W (n = 11)	M (n = 11)	Total (n = 22)	
DPPH [%]	48.99 (45.71–53.60)	46.56 (43.41–49.11)	48.69 (45.71–50.08)	48.88 (42.31–51.05)	46.25 (40.73–49.23)	44.80 (37.94–50.44)	0.238
DPPH [µM/L]	213.84 (203.17–228.85)	205.93 (195.66–214.23)	212.85 (203.17–217.39)	212.85 (192.10–220.55)	204.49 (186.9–214.63)	200.20 (177.87–218.58)	0.238
DPPH [mg/100mL]	5.53 (5.08–5.72)	5.15 (4.89–5.36)	5.33 (5.09–5.44)	5.33 (4.81–5.52)	5.13 (4.67–5.37)	5.01 (4.45–5.47)	0.238
FRAP [µM]	478.43 (414.83–693.42)	448.15 (390.61–757.00)	467.83 (414.84–693.41)	479.94 (429.98–669.19)	514.78 (448.1–723.69)	505.68 (437.23–635.88)	0.369
Phenols [mg/L]	41.53 (26.25–66.87)	76.62 (48.02–94.81)	56.31 (27.23–76.62)	60.75 (31.78–76.62)	74.99 (59.72–90.91)	74.35 (59.72–84.70)	0.022

Me—median; Q_25_—first quartile; Q_75_—third quartile; W—woman; M—man, n—number; *p*—level of statistical significance (Mann–Whitney U test between the study group and the total control group).

## Data Availability

The data supporting the findings of this study are available from the corresponding author upon request.

## Supplementary Materials

The following supporting information can be downloaded at: https://www.mdpi.com/article/10.3390/biology15100737/s1, Table S1: Correlation of Spearman’s rank order (*p* < 0.05) in the group of middle-distance runners without division by gender; Table S2: Correlation of Spearman’s rank order (*p* < 0.05) in the control group without division by gender; Table S3: Correlation of Spearman’s rank order (*p* < 0.05) in the control group without division by gender; Table S4: Correlation of Spearman’s rank order (*p* < 0.05) in the group of middle-distance runners without division by gender; Table S5: Correlation of Spearman’s rank order (*p* < 0.05) in the group of middle-distance runners without division by gender; Table S6: Correlation of Spearman’s rank order (*p* < 0.05) in the control group without division by gender.

## Abbreviations

ALAT	alanine aminotransferase
ASAT	aspartate aminotransferase
AI	Adequate Intake
BMI	Body Mass Index
DPPH	2,2-diphenyl-1-picrylhydrazyl
EAR	Estimated Average Requirement
FRAP	Ferric Reducing Antioxidant Power
GAE	Gallic Acid
Me	Median
Q_25_	first quartile
Q_75_	third quartile
RDA	Recommended Dietary Allowances
