# Supplementary material for: Nutritional Assessment of Polish Middle-Distance Runners: Analysis of Biochemical Parameters and Antioxidant Status—Pilot Study
Source: Biology (Basel). 2026 May 7;15(10):737. doi: 10.3390/biology15100737 (PMC13203526; doi:10.3390/biology15100737)
Supplement: Supplementary file 1 [file biology-15-00737-s001.zip › biology-4254455-supplementary.pdf]

Supplementary Materials

Table S1. Correlation of Spearman's rank order ($p < 0.05$) in the group of middle-distance runners without division by gender

	Age	Retinol	Vitamin A	β-Carotene	Vitamin E	Thiamine	Riboflavin	Niacin	Vitamin B6	Vitamin C	Folates	Panthotenic acid
Retinol	0.07		0.60	0.28	0.55	0.49	0.69	0.48	0.51	0.38	0.65	0.37
Vitamin A	0.15	0.60		0.81	0.64	0.57	0.68	0.72	0.65	0.76	0.77	0.66
β-Carotene	0.31	0.28	0.81		0.36	0.26	0.39	0.49	0.34	0.53	0.67	0.47
Vitamin E	0.03	0.55	0.64	0.36		0.68	0.70	0.53	0.54	0.54	0.67	0.63
Thiamine	-0.01	0.49	0.57	0.26	0.68		0.86	0.79	0.75	0.67	0.72	0.78
Riboflavin	0.08	0.69	0.68	0.39	0.70	0.86		0.73	0.69	0.69	0.79	0.72
Niacin	0.05	0.48	0.72	0.49	0.53	0.79	0.73		0.86	0.60	0.63	0.84
Vitamin B6	-0.16	0.51	0.65	0.34	0.54	0.75	0.69	0.86		0.54	0.68	0.69
Vitamin C	0.13	0.38	0.76	0.53	0.54	0.67	0.69	0.60	0.54		0.67	0.62
Folates	0.04	0.65	0.77	0.67	0.67	0.72	0.79	0.63	0.68	0.67		0.60
Panthotenic acid	0.28	0.37	0.66	0.47	0.63	0.78	0.72	0.84	0.69	0.62	0.60	
TPC	-0.14	0.35	0.43	0.49	0.07	-0.14	0.00	0.22	0.33	-0.10	0.28	-0.03
Number of meals	-0.04	0.24	0.48	0.52	0.38	0.29	0.19	0.22	0.22	0.24	0.56	0.30
Phenols intake	0.71	-0.15	0.10	0.33	-0.09	-0.13	0.00	-0.03	-0.26	0.11	-0.11	0.10
BMI	0.19	-0.05	-0.23	-0.21	-0.15	-0.20	-0.17	-0.04	-0.09	-0.45	-0.32	-0.11

correlations that were not presented in all the obtained results were not statistically significant ($p > 0.05$); statistically significant correlations are marked in red ($p < 0.05$); TPC – total phenols content in serum [mg/L]

Table S2. Correlation of Spearman's rank order ($p < 0.05$) in the control group without division by gender

	Age	Retinol	Vitamin A	β-Carotene	Vitamin E	Thiamine	Riboflavin	Niacin	Vitamin B6	Vitamin C	Folates	Panthotenic acid
Retinol	0.00		0.48	0.12	0.42	0.23	0.19	0.46	0.31	-0.16	0.43	0.49
Vitamin A	-0.19	0.48		0.88	0.46	0.28	0.17	0.52	0.60	0.06	0.44	0.38
β-Carotene	-0.15	0.12	0.88		0.20	0.03	0.01	0.26	0.40	0.09	0.25	0.35
Vitamin E	-0.26	0.42	0.46	0.20		0.66	0.59	0.74	0.53	0.29	0.65	0.37
Thiamine	-0.38	0.23	0.28	0.03	0.66		0.73	0.81	0.56	0.32	0.67	0.09
Riboflavin	-0.39	0.19	0.17	0.01	0.59	0.73		0.55	0.31	0.57	0.70	0.10
Niacin	-0.31	0.46	0.52	0.26	0.74	0.81	0.55		0.63	0.11	0.58	0.38
Vitamin B6	-0.30	0.31	0.60	0.40	0.53	0.56	0.31	0.63		-0.02	0.29	0.13
Vitamin C	-0.03	-0.16	0.06	0.09	0.29	0.32	0.57	0.11	-0.02		0.56	-0.18
Folates	-0.29	0.43	0.44	0.25	0.65	0.67	0.70	0.58	0.29	0.56		0.21
Panthotenic acid	-0.11	0.49	0.38	0.35	0.37	0.09	0.10	0.38	0.13	-0.18	0.21	
TPC	-0.38	-0.13	0.48	0.50	0.24	0.35	0.21	0.33	0.43	0.21	0.26	-0.05
Number of meals	0.09	0.20	0.40	0.21	0.44	0.49	0.55	0.28	0.37	0.22	0.40	0.05
Phenols intake	0.07	0.45	0.45	0.45	0.32	0.01	0.23	0.31	-0.04	0.42	0.51	0.55

DPPH	0.46	-0.15	-0.09	-0.09	-0.35	-0.26	-0.16	-0.27	0.07	-0.04	-0.40	-0.35

correlations that were not presented in all the obtained results were not statistically significant (p > 0.05); statistically significant correlations are marked in red (p < 0.05); TPC – total phenols content in serum [mg/L]; DPPH agreed as %I; age agreed as years

Table S3. Correlation of Spearman's rank order (p < 0.05) in the control group without division by gender

	Protein	Carbohydrates	Sodium	Potassium	Calcium	Phosphorus	Magnesium	Iron	Zinc	Copper	Manganese	Cholesterol	Fiber
Fat	0,662	0,647	0,534	0,553	0,536	0,644	0,700	0,654	0,688	0,708	0,160	0,516	0,577
Energy	0,867	0,919	0,637	0,732	0,682	0,874	0,841	0,806	0,863	0,762	0,351	0,487	0,754
Protein		0,811	0,622	0,574	0,633	0,858	0,679	0,625	0,844	0,552	0,196	0,549	0,579
Carbohydrate	0,811		0,587	0,792	0,682	0,894	0,851	0,816	0,808	0,752	0,394	0,299	0,799
Sodium	0,622	0,587		0,432	0,421	0,476	0,395	0,380	0,470	0,375	0,054	0,475	0,360
Potassium	0,574	0,792	0,432		0,618	0,813	0,840	0,912	0,677	0,842	0,583	0,228	0,889
Calcium	0,633	0,682	0,421	0,618		0,756	0,595	0,597	0,685	0,584	0,309	0,193	0,569
Phosphorus	0,858	0,894	0,476	0,813	0,756		0,880	0,857	0,897	0,781	0,492	0,285	0,817
Magnesium	0,679	0,851	0,395	0,840	0,595	0,880		0,930	0,845	0,946	0,539	0,199	0,869
Iron	0,625	0,816	0,380	0,912	0,597	0,857	0,930		0,787	0,935	0,584	0,231	0,935
Zinc	0,844	0,808	0,470	0,677	0,685	0,897	0,845	0,787		0,741	0,456	0,390	0,768
Copper	0,552	0,752	0,375	0,842	0,584	0,781	0,946	0,935	0,741		0,546	0,224	0,874
Manganese	0,196	0,394	0,054	0,583	0,309	0,492	0,539	0,584	0,456	0,546		0,058	0,673
Cholesterol	0,549	0,299	0,475	0,228	0,193	0,285	0,199	0,231	0,390	0,224	0,058		0,151
Fiber	0,579	0,799	0,360	0,889	0,569	0,817	0,869	0,935	0,768	0,874	0,673	0,151	

Riboflavin	-0,049	0,220	-0,125	0,311	0,091	0,163	0,303	0,438	0,188	0,432	0,206	0,229	0,396

correlations that were not presented in all the obtained results were not statistically significant ($p > 0.05$); statistically significant correlations are marked in red ($p < 0.05$);

Table S4. Correlation of Spearman's rank order ($p < 0.05$) in the group of middle-distance runners without division by gender

	Protein	Carbohydrates	Sodium	Potassium	Calcium	Phosphorus	Magnesium	Iron	Zinc	Copper	Manganese	Cholesterol	Fiber
Fat	0,716	0,777	0,730	0,638	0,596	0,706	0,714	0,790	0,699	0,743	0,400	0,444	0,606
Energy	0,900	0,973	0,864	0,800	0,706	0,877	0,779	0,844	0,825	0,792	0,569	0,471	0,761
Protein		0,879	0,918	0,749	0,758	0,943	0,691	0,747	0,852	0,679	0,518	0,504	0,774
Carbohydrate	0,879		0,814	0,803	0,710	0,866	0,752	0,808	0,801	0,774	0,619	0,447	0,766
Sodium	0,918	0,814		0,679	0,538	0,842	0,651	0,692	0,777	0,649	0,479	0,470	0,717
Potassium	0,749	0,803	0,679		0,578	0,818	0,912	0,879	0,857	0,905	0,818	0,492	0,951
Calcium	0,758	0,710	0,538	0,578		0,773	0,570	0,591	0,673	0,526	0,386	0,266	0,553
Phosphorus	0,943	0,866	0,842	0,818	0,773		0,805	0,816	0,899	0,786	0,635	0,497	0,866
Magnesium	0,691	0,752	0,651	0,912	0,570	0,805		0,912	0,910	0,965	0,847	0,345	0,947
Iron	0,747	0,808	0,692	0,879	0,591	0,816	0,912		0,860	0,930	0,651	0,548	0,877
Zinc	0,852	0,801	0,777	0,857	0,673	0,899	0,910	0,860		0,888	0,723	0,397	0,923
Copper	0,679	0,774	0,649	0,905	0,526	0,786	0,965	0,930	0,888		0,812	0,379	0,923

Manganese	0,518	0,619	0,479	0,818	0,386	0,635	0,847	0,651	0,723	0,812		0,171	0,858
Cholesterol	0,504	0,447	0,470	0,492	0,266	0,497	0,345	0,548	0,397	0,379	0,171		0,438
Fiber	0,774	0,766	0,717	0,951	0,553	0,866	0,947	0,877	0,923	0,923	0,858	0,438	
Thiamine	-0,374	-0,442	-0,268	-0,204	-0,399	-0,275	-0,182	-0,209	-0,312	-0,165	-0,116	-0,212	-0,123

correlations that were not presented in all the obtained results were not statistically significant ($p > 0.05$); statistically significant correlations are marked in red ($p < 0.05$);

Table S5. Correlation of Spearman's rank order ($p < 0.05$) in the group of middle-distance runners without division by gender

	Glucose [mg/dl]	Chlorides [mmol/l]	Calcium [mg/dl]	Magnesium [mg/dl]	Triglycerides [mg/dl]	Cholesterol [mg/dl]	Uric acid [mg/dl]	Iron [µg/dl]	ALAT [U/L]	ASAT [U/L]	FRAP
Chlorides [mmol/l]	0,101	1,000	0,353	0,451	0,005	-0,213	0,169	-0,613	-0,186	0,114	0,412
Calcium [mg/dl]	-0,209	0,353	1,000	-0,014	-0,406	-0,026	0,030	-0,564	-0,037	-0,057	0,353
Magnesium [mg/dl]	0,398	0,451	-0,014	1,000	-0,010	-0,520	0,635	-0,154	-0,242	0,195	0,574
Triglycerides [mg/dl]	-0,093	0,005	-0,406	-0,010	1,000	0,036	-0,235	0,174	-0,448	-0,283	-0,471
Cholesterol [mg/dl]	0,155	-0,213	-0,026	-0,520	0,036	1,000	-0,393	0,371	0,461	0,293	-0,146
Uric acid [mg/dl]	0,269	0,169	0,030	0,635	-0,235	-0,393	1,000	-0,053	0,179	0,188	0,637
Iron [µg/dl]	0,156	-0,613	-0,564	-0,154	0,174	0,371	-0,053	1,000	0,262	0,158	-0,295
ALAT [U/L]	0,058	-0,186	-0,037	-0,242	-0,448	0,461	0,179	0,262	1,000	0,381	0,096
Fat	0,228	0,049	0,194	0,460	-0,257	-0,222	0,079	-0,134	-0,168	-0,093	0,321

Copper	0,397	0,224	0,050	0,435	0,021	-0,075	0,035	-0,225	-0,084	0,028	0,165
Manganes	0,462	0,248	-0,049	0,326	0,251	-0,088	0,082	-0,330	-0,101	-0,103	0,094
Cholesterol	-0,124	0,168	0,481	0,181	-0,575	-0,131	0,348	-0,317	0,186	0,252	0,355
Vitamin A	-0,229	0,105	0,091	0,029	-0,086	0,276	-0,151	-0,104	0,199	0,462	0,046
Retinol	0,142	0,166	0,057	-0,040	-0,224	0,595	0,042	0,258	0,548	0,512	0,283
Thiamine	-0,020	0,333	-0,076	0,222	0,064	0,074	0,171	-0,083	0,229	0,468	0,037
Riboflavin	0,146	0,367	0,013	0,228	-0,105	0,354	0,007	0,082	0,245	0,617	0,253
Niacin	-0,143	0,202	0,082	0,179	-0,213	0,142	-0,003	-0,195	0,413	0,448	0,027
Vitamin B6	-0,108	0,012	-0,110	0,141	-0,114	0,208	0,148	0,043	0,524	0,693	0,040
Folates	-0,050	0,333	0,033	0,403	0,181	0,079	0,148	0,038	-0,078	0,477	0,202
Vitamin D	-0,044	0,224	-0,083	0,154	0,016	0,242	-0,062	0,123	0,239	0,601	0,060

correlations that were not presented in all the obtained results were not statistically significant ($p > 0.05$); statistically significant correlations are marked in red ($p < 0.05$);

Table . Correlation of Spearman's rank order ($p < 0.05$) in the control group without division by gender

	Total Protein [g/dl]	Albumin [g/dl]	Chlorides [mmol/l]	Calcium [mg/dl]	Magnesium [mg/dl]	Phosphorus [mg/dl]	Iron [µg/dl]	ALAT [U/L]	ASPAT [U/L]	DPPH	FRAP
Glucose [mg/dl]	0,228	0,419	0,389	-0,150	-0,215	0,045	-0,190	0,088	0,510	0,215	-0,252
Total protein [g/dl]		0,481	0,038	-0,235	-0,164	0,049	-0,197	-0,129	-0,057	0,602	-0,181
Albumine [g/dl]	0,481		0,524	-0,068	-0,143	0,354	-0,068	0,023	0,261	0,445	-0,233
Chlorides [mmol/l]	0,038	0,524		0,027	0,149	0,622	0,197	-0,056	-0,007	-0,203	-0,085
Calcium [mg/dl]	-0,235	-0,068	0,027		-0,039	0,208	0,063	-0,020	-0,127	0,029	0,539

Magnesium [mg/dl]	-0,164	-0,143	0,149	-0,039		0,064	0,149	-0,063	-0,165	-0,459	0,436
Cholesterol [mg/dl]	0,128	0,208	-0,243	-0,093	0,027	-0,112	-0,333	0,339	0,453	0,185	-0,039
ALAT [U/L]	-0,129	0,023	-0,056	-0,020	-0,063	0,357	-0,371	1,000	0,604	-0,165	0,150
DPPH	0,602	0,445	-0,203	0,029	-0,459	-0,261	-0,302	-0,165	0,132	1,000	-0,066
FRAP	-0,181	-0,233	-0,085	0,539	0,436	-0,068	0,001	0,150	-0,206	-0,066	1,000
Pholiphenols	0,094	0,442	0,352	-0,143	0,260	0,368	0,145	0,085	-0,011	-0,047	0,074
Calcium	0,178	-0,075	-0,129	-0,109	-0,110	-0,145	-0,518	-0,002	-0,225	-0,104	0,029
Vitamin E	-0,252	-0,064	0,250	-0,200	0,001	0,399	0,220	0,517	0,173	-0,345	0,063
Niacin	-0,252	-0,114	0,136	-0,248	0,033	0,418	0,146	0,581	0,280	-0,272	0,090
Folates	-0,284	-0,298	0,052	-0,248	0,001	0,133	0,450	0,095	-0,205	-0,402	-0,130
Vitamin D	-0,499	-0,159	0,044	-0,005	-0,084	0,186	0,461	0,218	-0,033	-0,222	0,166

correlations that were not presented in all the obtained results were not statistically significant ($p > 0.05$); statistically significant correlations are marked in red ($p < 0.05$);
